# Quantitative Muscle MRI and Clinical Findings in Women With Pathogenic Dystrophin Gene Variants

**DOI:** 10.3389/fneur.2021.707837

**Published:** 2021-09-03

**Authors:** Freja Fornander, Tuva Åsatun Solheim, Anne-Sofie Vibæk Eisum, Nanna Scharff Poulsen, Annarita Ghosh Andersen, Julia Rebecka Dahlqvist, Morten Dunø, John Vissing

**Affiliations:** ^1^Department of Neurology, Copenhagen Neuromuscular Center, Rigshospitalet, University of Copenhagen, Copenhagen, Denmark; ^2^Department of Clinical Genetics, Department of Neurology, Rigshospitalet, University of Copenhagen, Copenhagen, Denmark

**Keywords:** dystrophinopathy, female carriers, Dixon MRI, muscle fat infiltration, dynamometry, muscle strength

## Abstract

**Objective:** To explore fat replacement, muscle strength, and clinical features in women heterozygous for a pathogenic *DMD* variant, we prospectively examined 53 women, assuming that some of these women—despite of the recessive X-linked inheritance—manifested clinical symptoms.

**Methods:** We performed a cross-sectional observational study using MRI and stationary dynamometry of lower extremities, extracted blood muscle biomarkers, and investigated subjective complaints. Results were compared with 19 healthy women.

**Results:***DMD* variant carriers were weaker and had higher fat fractions than controls in all investigated muscle groups (*p* < 0.02). Fat fractions were 18% in carriers vs. 11% in controls in thighs (*p* = 0.008), and 15 vs. 11% in calf muscles (*p* = 0.032). Seventy-two percent had fat fractions deviating from controls by two standard deviations (SDs) in one or more of the 16 investigated muscle groups. On strength testing, 40% of the carriers had results deviating from control muscle strength by two SDs in one or more dynamometry assessments. Forty-three carriers (81%) had either reduced muscle strength (<2 SDs from control mean) and/or elevated muscle fat fraction (>2 SDs from control mean). Thirty of these had subjective symptoms. Blood creatine kinase and myoglobin were elevated in 57% of the carriers.

**Conclusion:** Using quantitative methods, this study shows that both clinically symptomatic and asymptomatic women with pathogenic *DMD* variants show a high prevalence of muscle affection. Longitudinal studies in female carriers of pathogenic *DMD* variants are needed to follow the evolution of these changes.

## Introduction

Duchenne (DMD) and Becker (BMD) muscular dystrophies are two of the most common inherited disorders of muscle, caused by X-linked recessively inherited mutations in the dystrophin gene (*DMD*) (1, 2). The cytoskeletal protein dystrophin is needed for signaling and stabilizing the muscle cell structure, and if absent or decreased, it eventually leads to cell necrosis and replacement by fat and connective tissue (2).

Males are primarily affected, because of the X-linked inheritance. Approximately one in 5,000 boys are born with pathogenic *DMD* mutations (3, 4). The point prevalence of DMD is approximately 8.3/100,000 and 7.3/100,000 for BMD (5), making them two of the most common inherited disorders of muscle. Two-thirds of the pathogenic variants in affected boys are inherited from their mother, and one third are caused by *de novo* variants (2).

It is well-known that female carriers of a pathogenic *DMD* variant can be affected. In these carriers, the reported prevalence of muscular and/or cardiac symptoms is highly variable with rates of 2.5–84.3% (6–13). A prevalence of 0.43/100,000 manifesting female carriers was reported in Northern England (5).

Patients with dystrophinopathies experience progressive, proximal muscle weakness and atrophy, respiratory insufficiency, and cardiomyopathy. The symptoms are most severe in males with DMD, resulting in loss of ambulation usually before age 12 years. In BMD, the phenotype has a milder and wider spectrum of severity (1). In manifesting women, symptoms have been reported as ranging from mild myopathy and cardiomyopathy to DMD-like phenotypes, but most *DMD* variant carriers reportedly have no complaints or signs of muscle weakness (8, 11, 14, 15).

Skeletal muscle MRI is increasingly used as a tool to diagnose and characterize neuromuscular disease. In most muscular dystrophies, there is muscle atrophy and replacement of muscle tissue by fat (2). MRI can illustrate and quantify both muscle volume, cross-sectional area, and fat replacement of muscle (16–19).

Our objective with this study was to study potential muscle involvement in women with pathogenic *DMD* variants using a prospective, cross-sectional design, using quantitative assessments of MRI-based muscle fat fraction and muscle strength measures. By combining MRI and muscle strength results, it allowed us also to investigate muscle contractile properties.

## Materials and Methods

### Standard Protocol Approvals and Patient Consents

All participants gave their informed oral and written consent. Data handling and protocols were approved by the Danish Data Protection Agency and the National Committee of Health Research (H-16035677).

### Subjects and Controls

Subjects were recruited *via* the Copenhagen Neuromuscular Clinic and Department of Clinical Genetics at the national hospital, Rigshospitalet. Invitations were sent by letter and included information about the study. Inclusion criteria were (i) women who were confirmed carriers of pathogenic variants in the *DMD* gene, and (ii) minimum 18 years of age for ethical approval reasons and because our department only sees adults. Exclusion criteria were contraindications for MRI (e.g., metal implants, pregnancy, claustrophobia).

Nineteen healthy women were used as controls to compare muscle fat fraction and strength in *DMD* variant carriers. The controls, who participated in a previous study (20) were recruited *via* the local community and investigated 2 years prior to the investigations of the female *DMD* variant carriers. MRIs in *DMD* variant carriers were performed and analyzed by F.Fo., while MRIs in healthy controls were performed and analyzed by J.R.D.

### Genetic Testing

Subjects were identified, previous to the study, by segregation analysis performed in relation to a male relative with a confirmed dystrophinopathy. DNA was isolated from an EDTA blood sample by a standard method. Male probands were initially assessed with multiplex ligation-dependent probe amplification (MLPA) for detection of exonic deletions/duplications. If no quantitative aberration was detected, the entire coding and exon-flanking sequence of *DMD* was sequenced by a targeted next-generation sequencing approach (NGS) to a minimum depth of 100× in a clinical setting. Subsequent segregation analysis was performed through a mutation-specific analysis.

### Assessments

Subjects underwent MRI, manual strength examination, dynamometry testing of lower-extremity muscles, and blood sampling for measurements of creatine kinase and myoglobin and were asked to report about subjective experiences of pain, weakness, and physical function. They also underwent cardiac investigations presented in an accompanying article in *Frontiers in Neurology*. Our primary outcomes presented here were lower-extremity muscle fat fraction and strength.

### MRI Evaluations

**Figure 1:**
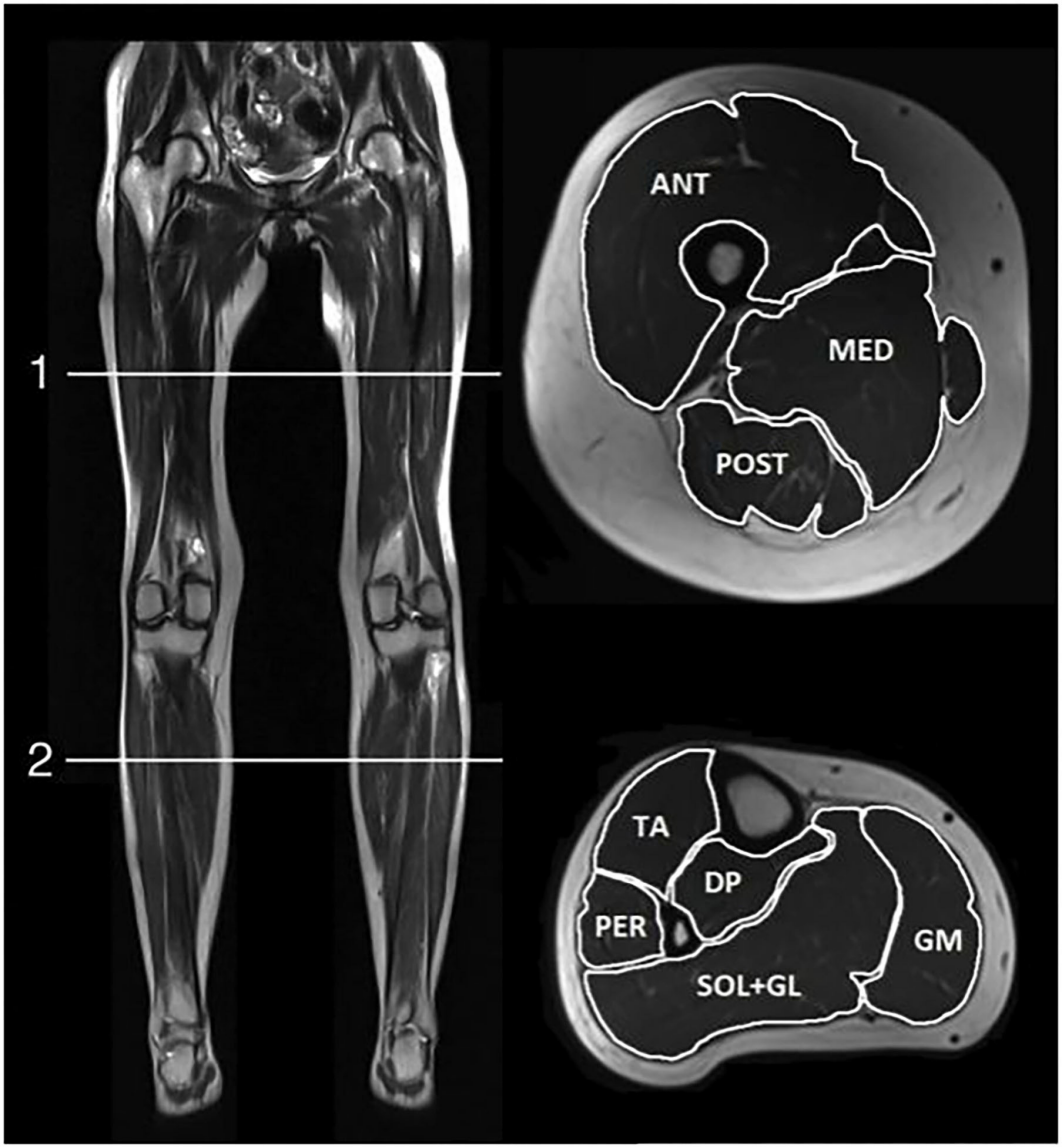
MRI scan positions. T1-weighted MRI images of scan positions (left) and muscle groups outlined in cross sections of thigh (top right) and lower leg (bottom right). ANT, anterior thigh muscle compartment; MED, medial thigh muscle compartment; POST, posterior thigh muscle compartment; AC, anterior calf muscle compartment; PER, peroneal muscles; DP, deep posterior calf muscle compartment; SOL+GL, soleus and lateral gastrocnemius muscles; GM, medial gastrocnemius muscle.

We used a 3.0T Siemens (Erlangen, Germany) MAGNETOM Verio Tim System. Participants were positioned supine with a peripheral angio coil over their legs and a matrix coil over the pelvis. From three-dimensional localizers, T1-weighted and Dixon sequence images were taken. The same MRI protocol was used for both controls and carriers. It included axial T1-weighted images (field of view = 400–450 mm, slice thickness = 6.0 mm, distance factor = 20%, echo time = 19 ms, repetition time = 650 ms) and axial two-point Dixon sequences (field of view = 400–450 mm, slice thickness = 3.5 mm, distance factor = 0%, echo time = 2.45 and 3.675 ms, repetition time = 5.59 ms). Slices used for muscle segment outlining were acquired at 50% of the length of femur and 33% of the length of tibia. It has been established that the use of a single, cross-sectional slice is a representative basis for total muscle volume and fat infiltration calculations (21). To ensure that fat replacement was not a focal phenomenon, all T1-weighted scans were visually controlled at multiple slices. Fat fraction (FF) in the carriers was calculated using *Osirix Lite* (Pixmeo, Switzerland) and *Horos* (The Horos Project, Annapolis, MD, USA) MRI imaging software by manually segmenting three groups of the thigh (short head of biceps femoris excluded due to technical difficulties to outline the muscle) and five groups of the calves (Figure 1). Controls' FF was calculated in Siemens Syngo MR Workplace using Numaris/4 B17 software (Siemens AG, Munich, Germany). When outlining the muscle groups, visible fat around blood vessels, nerves, bone, and the peripheral muscle borders was excluded and the cross-sectional area and signals from fat and water were determined. Muscle FF was calculated: FF (%) = (signal fat/signal fat + water) × 100. Muscle FF was considered abnormal when it was higher than control mean + 2 standard deviations (SD). We calculated the fat-free, or contractile, cross-sectional area (CCSA, cm^2^): cross-sectional area × (1—fat fraction). Interrater variability analysis was performed in five healthy controls. Anterior, medial, and posterior muscle groups of the right and left thighs were outlined by F.Fo. and J.R.D, and differences in fat fractions were compared.

### Muscle Strength Testing

A Biodex System 4 Pro (Biodex Medical Systems, Shirley, NY, USA) stationary dynamometer was used to measure lower-extremity maximum strength. Subjects were fixed in the chair in accordance with *Biodex Multi-Joint System Pro* guidelines (22) (Biodex Medical Systems, Inc., USA.), and the tests included isokinetic knee flexion and extension [80° range of motion (ROM)], and ankle dorsi- and plantar flexion (48° ROM, with knee fixed at a 120 to 130° angle, with shoes off). Four investigators carried out the strength testing for the carriers and 1 for the controls. All investigators were instructed in the correct use of the Biodex according to the guidelines. A standardized prerecorded voice instructor was used during the testing of all participants. The women were instructed to use maximum strength at full extension and flexion every 15 s for eight repetitions per joint. The peak torque values [expressed in Newton meters (Nm)] were used for analysis. Bilateral measurements were performed in order to evaluate any presence of asymmetry. Control strength data were unilateral, and comparison of the specific muscle force between carriers and controls was therefore calculated for the right leg only. Dynamometry measurements were considered abnormal when they were two standard deviations (SD) lower than control mean. After muscle dynamometry, all subjects were tested manually for muscle strength according to the Medical Research Council scale (MRC) (23) in knee and ankle extension and flexion.

### Specific Muscle Force

To investigate the contractile properties in *DMD* variant carriers, the peak torque per fat-free muscle volume (pT/CCSA) was calculated and compared to findings in controls.

### Secondary Clinical Assessments

All subjects were asked to report if they had experienced any myalgia/muscle pain or limb weakness, according to their own perception. When questioned about daily physical activity and function, the participants were asked about their ability to run or walk up a flight of stairs. They were categorized as (0) cannot manage stairs, (1) can only climb stairs with either personal assistance or banisters, (2) can walk, but not run up a flight of stairs without the use of banisters/assistance, and (3) no affection—can run/hurry up a flight of stairs without any assistance. Also, all subjects completed the self-administered Fatigue Severity Scale (FSS) (24), which is well-validated for many chronic diseases and in healthy controls (24, 25). Here, the participants answered nine questions about the subjective experience of fatigue, each question scored from one to seven points, and a mean score from all questions was used as the total FSS score.

### Statistical Analysis

Statistics were carried out using Microsoft Excel, SPSS, and R. Quantitative values are presented as mean ± standard deviations. To calculate significant differences between variant carriers and controls, between carriers with DMD-associated vs. BMD-associated variants, and between right and left sides, *Student's t-tests* and repeated-measure ANOVA were used. When there was a significant main effect, *post hoc* pairwise comparisons were performed. Pearson correlation and linear regression were performed to test correlation. Univariate ANOVA was performed to test the difference between slopes. Chi^2^ was used when comparing groups of categorical values. The level of statistical significance was set to 0.05. A Bland–Altman plot was used for interrater variability analysis and the ICC (intraclass correlation coefficient) was calculated, using R-packages BlandAltmanLeh, ICC, and irr.

## Results

### Inclusion and Demographics of *DMD* Variant Carriers for the Study

A total of 111 known *DMD* variant carriers were invited to join the study. Fifty-three subjects (mean age 49.6 years; range 26–81), originating from 45 families, accepted to participate and fulfilled the inclusion criteria. All 53 were genetically verified carriers of pathogenic *DMD* variants, and all had relatives diagnosed with either DMD or BMD, except for three sporadic *de novo* variant carriers who had *DMD* variants most likely to cause a Duchenne phenotype in men and were categorized as such in this study. Of the 53 participants, 33 had *DMD* variants predicted to produce a Duchenne phenotype in men (DMD-associated), and 20 a Becker phenotype in men (BMD-associated). These assumptions were based on male relatives' diagnosis or predicted pathogenicity according to database records in the three participants with no affected male relatives. The DMD- and BMD-associated groups were comparable for age (50.3 vs. 48.6 years) and BMI (25.6 vs. 27.4 kg/m^2^). One of the participants only completed unilateral testing, due to recent knee surgery. Calf MRI scans of one other participant was excluded due to image processing artifacts. Three participants could not do ankle strength testing on one side due to recent ankle injuries and therefore only contributed with unilateral data. The 19 healthy controls were aged 46.5 years (range: 26–64) and had a BMI of 22.9 (range 19–28.6 kg/m^2^). Due to technical problems, dynamometry data from one control were excluded.

Fifty-eight women were not included in the study due to either meeting exclusion criteria (*n* = 1), not wishing to participate (*n* = 19), or not responding to our letter and phone call (*n* = 38). To uncover potential selection bias, the excluded women were contacted by telephone after completion of the study for short interviews on their reasons not to participate and presence of muscular symptoms. Thirty-eight women could not be reached. Twenty women responded, and reasons for not participating were reported as distance to research site too far/lack of time (*n* = 10); too many hospital visits for other reasons (sick child/family member) or own non-*DMD*-related illness (*n* = 6); had MRI contraindications (*n* = 1); and never received the invitation letter (*n* = 3). One of these did not wish to give information about symptoms, but just reported she never received an invitation. No one reported muscular symptoms as reasons for rejection. There was no significant difference in reported experience of symptoms (muscle weakness, muscle pain, or fatigue): Forty-seven percent of participants vs. 31.5% of the 19 fully interviewed non-participants reported symptoms (*p* = 0.24).

### Muscle Strength

**Figure 2:**
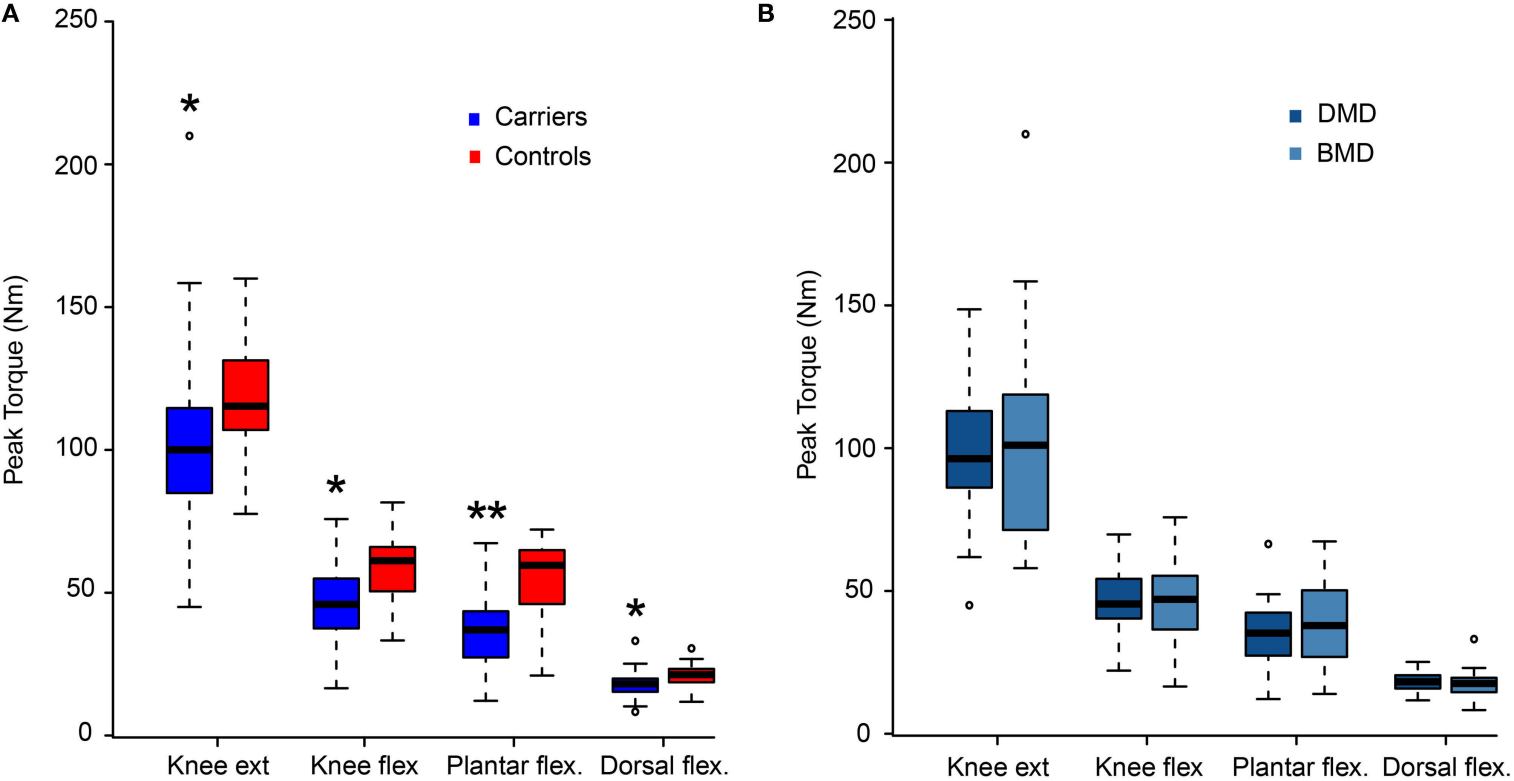
Dynamometry results. Maximum strength presented as peak torque comparing women with pathogenic variants in *DMD* and healthy women (Controls) **(A)**, and comparison between women with variants predicted to result in Duchenne muscular dystrophy (DMD) and Becker muscular dystrophy (BMD) **(B)**. Presented as median value and four quartiles. ext, extension; flex, flexion; Nm, Newton meter; *p < 0.05 and **p < 0.001 vs. controls.

Muscle strength, measured by isokinetic dynamometry, was lower in thigh and calf muscles of *DMD* variant carriers compared with controls (*p* = 0.001; Figure 2). Twenty-one women (40%) had peak torque deviating from values in controls by two SDs or more in one or more dynamometry exercises. These 21 women were 54.2 years old (range 29–81) [control mean age 46.5 (range: 26–64)]. Peak torque did not differ between carriers of DMD- and BMD-associated variants (*p* = 0.36). When comparing strength in the left and right legs in variant carriers, no overall significant strength difference was found (*p* = 0.75). However, six subjects did show a notable asymmetry of more than 50% weaker performance contralaterally in at least one of the four strength assessments. No participants scored below 4- on the MRC scale. Nine women scored 4-, 4, or 4+ (moderate weakness) in at least one test. The remaining 44 women scored 5 in all tests.

### Specific Muscle Force

**Figure 3:**
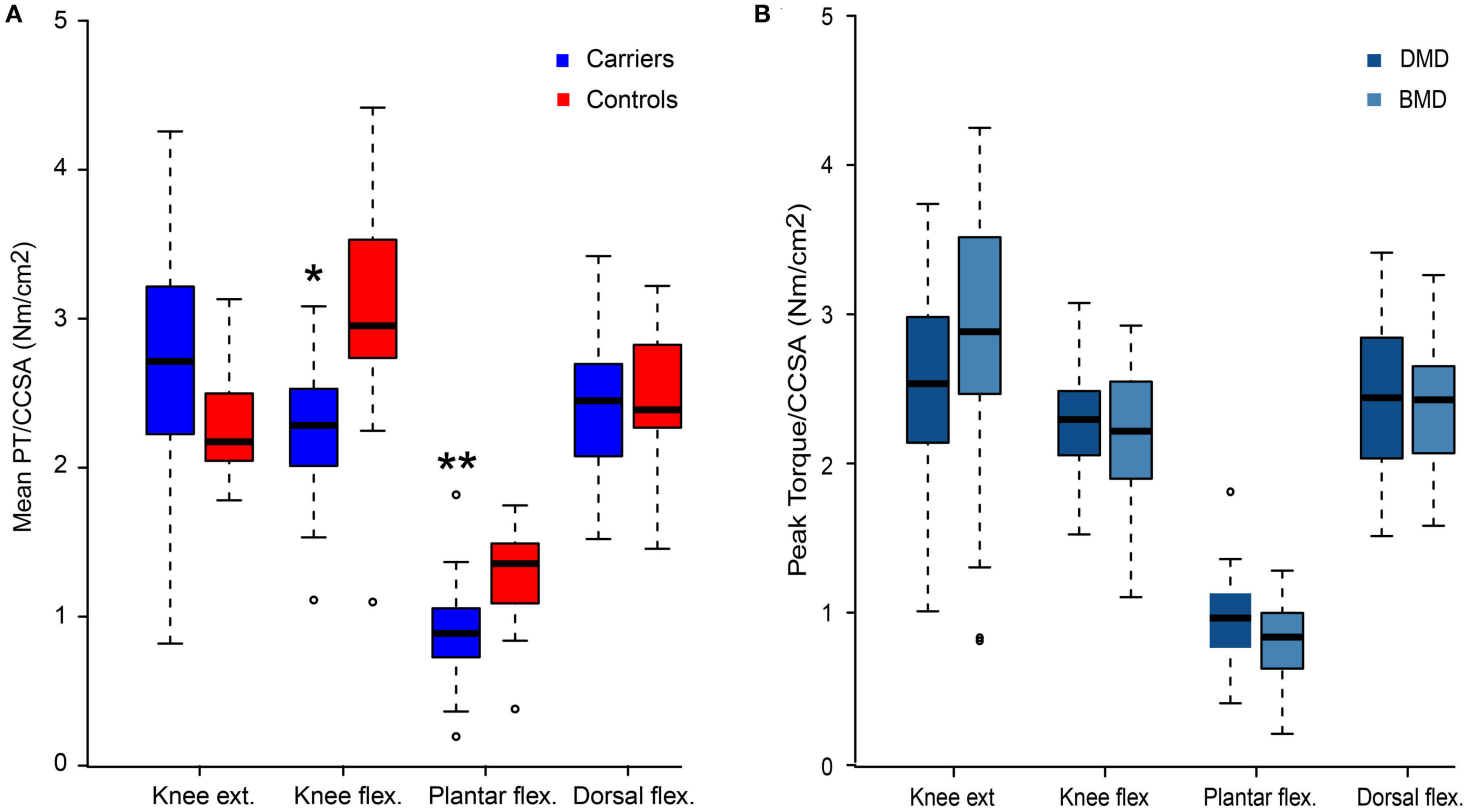
Peak torque/contractile cross-sectional area. Peak torque/CCSA values for women with pathogenic variants in *DMD* compared to healthy women (Controls) **(A)**, and comparison of peak torque/CCSA values in women with variants predicted to result in Duchenne muscular dystrophy (DMD) and Becker muscular dystrophy (BMD) **(B)**. Presented as median value and four quartiles. CCSA, contractile cross-sectional area; ext, extension; flex, flexion; *p < 0.05 and **p < 0.001 vs. controls.

The specific muscle force (pT/CCSA) was decreased compared to controls (main effect *p* = 0.008, Figure 3A). *Post-hoc* pairwise comparisons showed decreased strength in both knee and plantar flexion (*p* = 0.012 and *p* < 0.0001, respectively) but not in knee extension and ankle dorsal flexion. The specific muscle force did not differ between DMD- and BMD-associated carriers (*p* = 0.57, Figure 3B). With increasing peak torque, a correlating linear increase in CCSA was seen in all four strength analyses (knee extension: *r* = 0.83, knee flexion: *r* = 0.53, plantar flexion: *r* = 0.48, dorsal flexion; *r* = 0.64; *p* < 0.001). When we calculated this relation for the 18 controls, the correlation coefficients were not significant.

### Muscle Fat Fraction (MRI)

**Figure 4:**
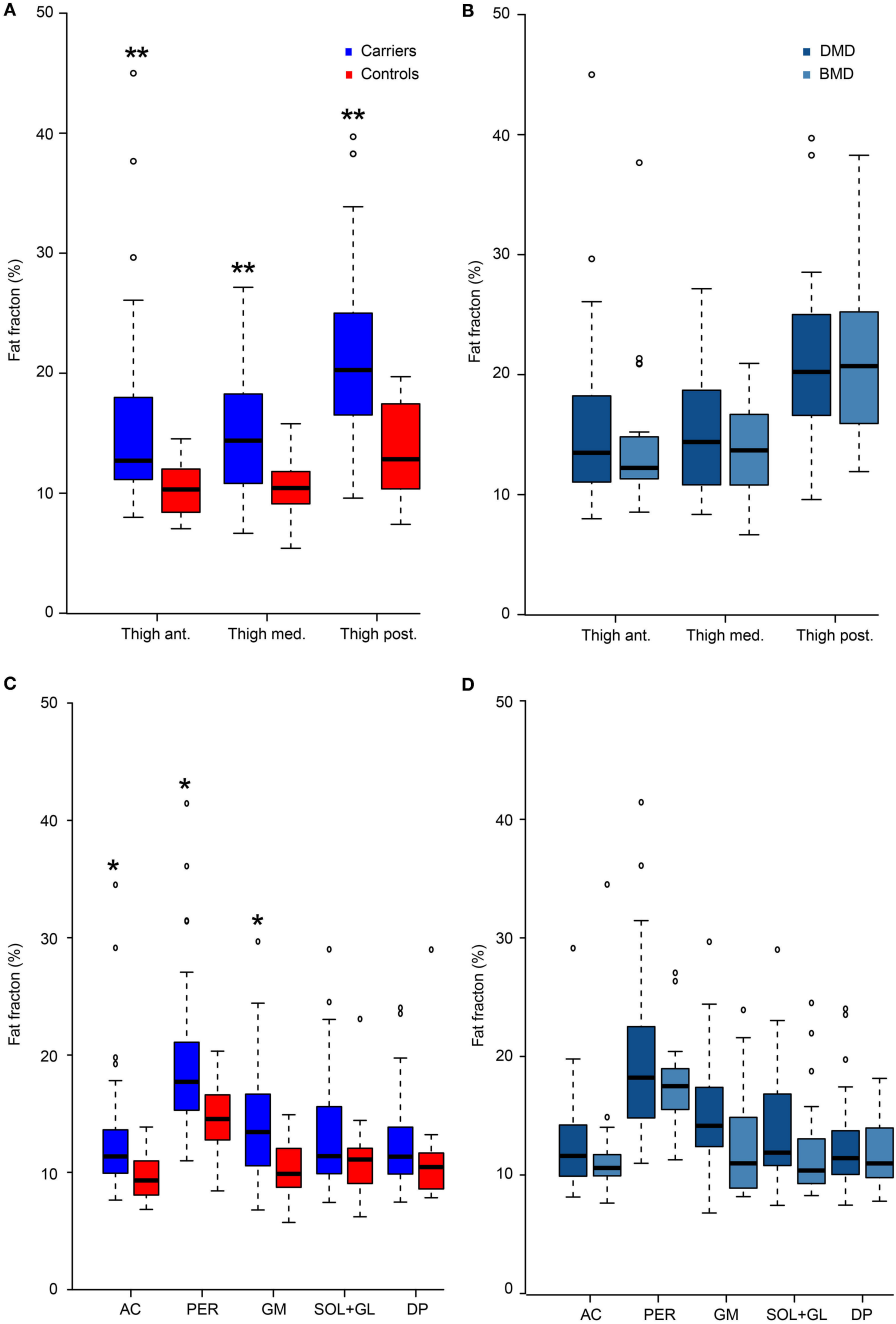
Fat fraction. Fat fractions in women with pathogenic variants in *DMD* compared to healthy women (controls) in thigh **(A)** and calf muscles **(C)**, and comparison of fat fractions in women with variants predicted to result in Duchenne muscular dystrophy (DMD) and Becker muscular dystrophy (BMD) in thigh **(B)** and calf **(D)** muscles. Presented as median value and four quartiles. Ant, anterior thigh muscles; Med, medial thigh muscles; Post, posterior thigh muscles; AC, anterior calf compartment; PER, peroneal muscle compartment; GM, medial gastrocnemius muscle; SOL+GL, soleus and lateral gastrocnemius muscle; DP, deep posterior calf muscles. *p < 0.05 and **p < 0.001 vs. controls.

**Figure 5:**
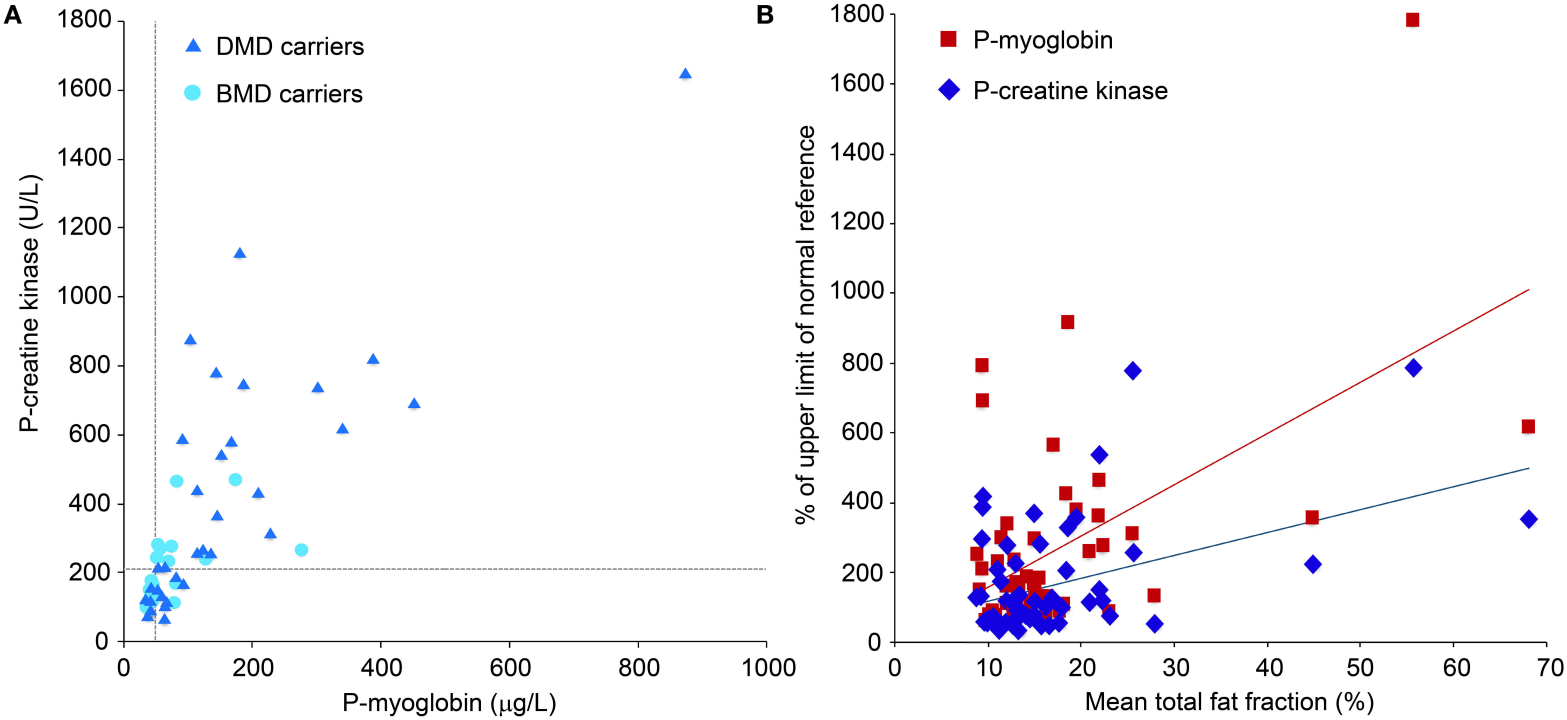
Biomarkers. Plasma creatine kinase (CK) and myoglobin levels from blood samples in women with pathogenic variants in the *DMD* gene predicted to cause Duchenne muscular dystrophy (DMD) and Becker muscular dystrophy (BMD). Reference cutoffs are marked with light blue lines **(A)**. Normal CK reference level 35–210 U/l, normal myoglobin reference level 19–49 μg/L. **(B)** shows the correlation between mean lower-extremity fat fractions and the two different plasma biomarkers creatine kinase and myoglobin (*R* = 0.53 and *R* = 0.612, respectively; *p* < 0.001). To be comparable, CK and myoglobin are presented as % of upper limit of normal.

Muscle FF was higher in *DMD* variant carriers than in controls (*p* = 0.014; Figure 4). Thirty-eight of the carriers (72%) had abnormally high FFs (>2SDs of control mean) in at least one of the 16 lower-extremity muscles or muscle groups. Ten of these 38 women had abnormal FF in as many as 10 or more muscles/muscle groups. No difference in total FF was found between DMD- and BMD-associated *DMD* variant carriers (*p* = 0.39). In *DMD* variant carriers, there was asymmetry in FF between the two legs (*p* = 0.016, Figure 5), with no difference in asymmetry between DMD- and BMD-associated variant carriers (*p* = 0.675). Controls had no FF asymmetry between sides (*p* = 0.59).

#### Thighs

The bilateral mean values of the dystrophinopathy carriers' FFs of the anterior, medial, and posterior groups were all higher than in controls (*p* < 0.001). Furthermore, all these muscle groups had abnormally high mean FF (>2SDs of control mean). Significant asymmetry was found in the posterior muscle group (*p* < 0.001) of *DMD* variant carriers. Six women showed asymmetry of more than 10% points in one or more thigh muscle groups. The mean composite FF of all three muscle groups in thighs was 18% (SD 11.8) compared to 11% (SD 2.6) in controls (control mean + 2SD = 16.1%).

#### Calves

The bilateral mean FF of the five calf muscle groups was higher than control mean FF (main effect, *p* < 0.03). However, FF was not significantly different from healthy women in the soleus and lateral gastrocnemius muscles (SOL + GL) and deep posterior compartment (DP) in the *post-hoc* pairwise comparison (*p* = 0.16 and *p* = 0.31, respectively). Statistically significant asymmetry was found in the peroneal muscles (PER) (*p* < 0.001) and the SOL+GL (*p* = 0.034), but not in the other three calf muscle groups (AC, GM, DP) of *DMD* variant carriers. Asymmetry of more than 10 percentage point difference in FF between sides was seen in eight women, predominantly in PER. The mean composite FF of all five muscle groups in calves was 15.2% (SD 10) compared to controls at 10.9% (SD 2.8) (control mean + 2SD = 16.5%).

#### Interrater Variability Analysis

The Bland–Altman plot showed 2 outliers out of 32 observations (6 %). All observations were scattered randomly throughout the plot; hence, no skewed pattern was seen. Differences were normally distributed. Mean differences were−1.09 with the 2SD upper limit being 2.77 and the 2SD lower limit being−4.95. The ICC was 0.896 (CI 0.504;0.984), indicating a good correlation.

### Clinical Assessments/Secondary Outcomes

#### Plasma CK and Myoglobin

**Figure 6:**
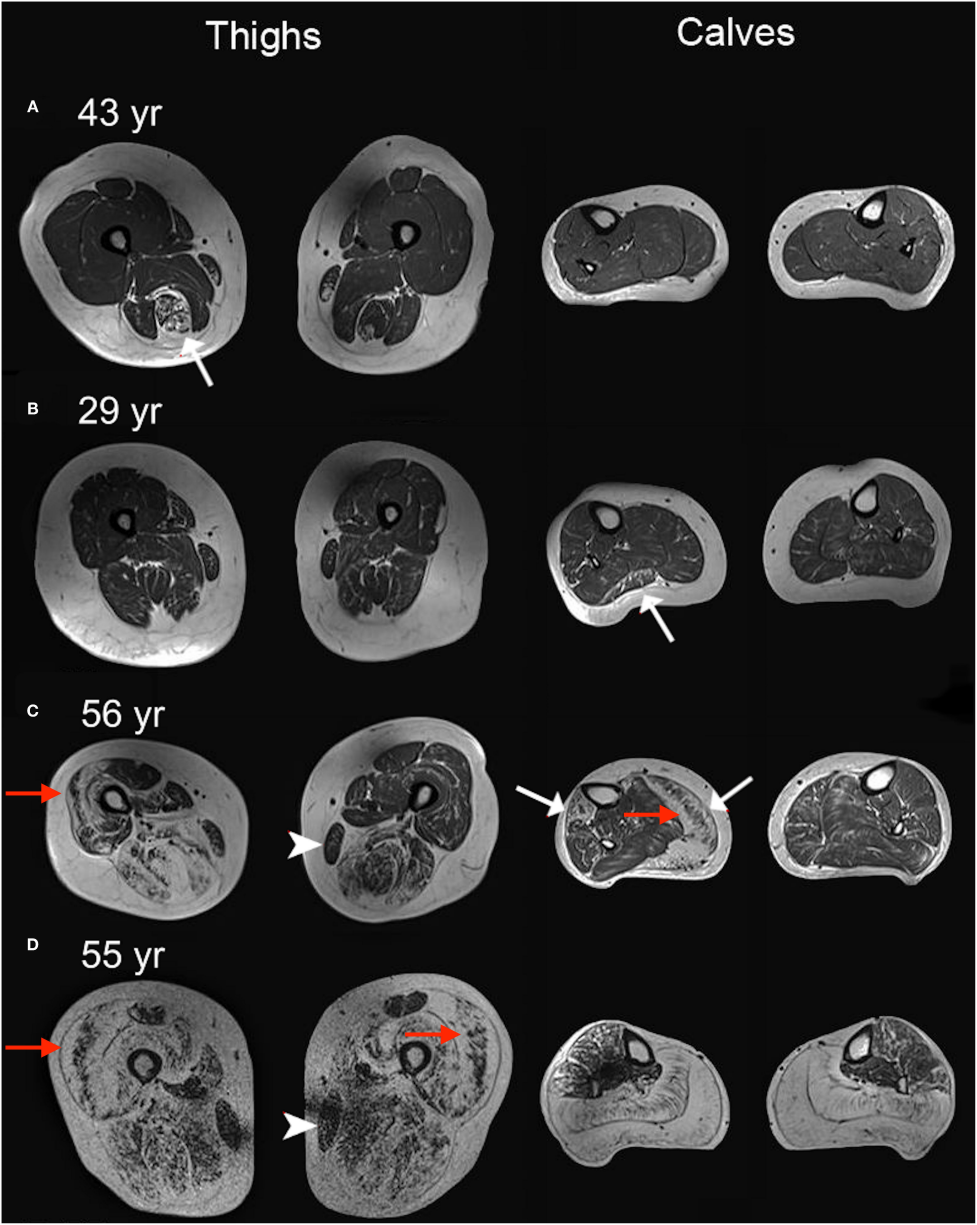
Four T1-weighted examples. T1-weighted MRI images of thighs and calves of four women heterozygote for pathogenic *DMD* variants. White represents fat tissue. **(A–D)** shows a range of muscle affection from mild to severe. Cases **(C,D)** are particularly affected with prominent replacement of muscle by fat. Correspondingly, muscle strength was compromised in these patients. Cases **(A–C)** have asymmetric fatty replacement (white arrows) in one or more muscles. Note the sparing of the gracilis muscle in cases **(C,D)** (arrowheads). Sandwich sign (preserved core of muscle surrounded by fat replaced muscle), reminiscent of what is seen in Bethlem myopathy, is observed in several muscles (red arrows). For more details on these four cases, see Table 1: subject no. 3 for **(A)**; subject no. 34 for **(B)**; subject no. 23 for **(C)**; subject no. 32 for **(D)**.

CK was above the normal reference level in 30 (57%) *DMD* variant carriers. Myoglobin was above the normal reference level in 39 (73%) *DMD* variant carriers (Figure 5). CK and myoglobin correlated positively with the composite total mean FF (*R* = 0.53 and *R* = 0.612, respectively; *p* < 0.001; Figure 6B). DMD-associated carriers had higher mean CK (425 U/l; SD 360) than BMD-associated carriers (209 U/l; SD 110; *p* = 0.003). Mean myoglobin was 127 μg/l (range 34–873 μg/l), and DMD-associated carriers had higher levels than BMD-associated carriers (159 μg/l; SD 165, vs. 76 μg/l; SD 13, *p* = 0.012).

**Table 1:** Demographics and characteristics of the 53 women with pathogenic variants in the dystrophin gene.

**Subject**	**Age (years)**	**Predicted phenotype**^†^****	**CK**^††^** (U/L)**	**Self-reported weakness. pain or fatigue **^†††^****	**Age at first symptom**	**Age at verified carrier status (yrs)**	**Physical function (score)**^††††^****	**FSS**^†††††^** (score)**	**Pathogenic *DMD* variants^♦^**	**Mean fat fraction thighs (%)^♦♦^**	**Mean fat fraction calves (%)^♦♦♦^**	**Number of abnormal muscles^♦♦♦♦^**	**Leg dynamometry performance compared to controls**^♦♦♦♦♦^****
1	61	DMD	783	No	N/A	46	3	1.8	Del ex. 3–16 (in-frame)	15.6	13.5	2	Normal
2	60	DMD	693	No	N/A	52	3	3.6	Dup ex. 2 (out-of-frame)	21.5	12.8	8	Normal
3	43	BMD	98	W	Childhood	41	3	3.6	Del ex. 3-9 (in-frame)	18.8	11.1	4	Normal
4	67	DMD	432	No	N/A	59	3	2.4	Dup ex. 2 (out-of-frame)	21.1	13.4	8	Normal
5	36	DMD	214	No	N/A	19	3	4.3^*^	Del ex. 45-52 (out-of-frame)	19.6	15.3	9	Decreased
6	39	DMD	820	No	N/A	26	3	1.8	Del ex. 45-50 (out-of-frame)	9.4	N/A	0	Normal
7	26	DMD	370	No	N/A	24	3	2.8	c.3806delA	11.0	12.0	1	Normal
8	62	DMD	168	No	N/A	37	3	3.0	c.2622+1G->A	15.4	11.7	3	Decreased
9	65	DMD	581	W. P	Childhood	59	2	5.8^*^	Del ex. 3-41 (in-frame)	12.1	11.6	0	Decreased
10	30	BMD	151	W. P	28 yrs	29	3	2.4	Del ex. 48 (in-frame)	10.7	9.2	0	Decreased
11	56	DMD	89	No	N/A	50	3	2.6	Del ex. 44 (out-of-frame)	13.6	10.8	0	Normal
12	70	DMD	213	W	68 yrs	69	1	4.6^*^	Del ex. 46-55 (out-of-frame)	16.6	15.2	4	Decreased
13	54	BMD	177	W	40 yrs	-	1	4.8^*^	c.2434T>A; p.(Trp812Arg)	24.6	20.3	14	Decreased
14	60	DMD	315	No	N/A	44	3	1.2	Del ex. 45-52 (out-of-frame)	25.2	17.7	11	Normal
15	48	BMD	466	P	45 yrs	42	3	1.7	c.10368delT	14.4	10.8	2	Normal
16	66	BMD	264	No	N/A	28	3	2.0	Del ex. 51 (out-of-frame)	17.4	15.9	5	Normal
17	64	BMD	132	No	N/A	51	3	3.6	Del ex. 37-43 (out-of-frame)	16.1	11.7	2	Decreased
18	43	DMD	590	W	24–29 yrs	41	3	2.6	Del ex. 52 (out-of-frame)	16.2	14.1	3	Decreased
19	47	DMD	157	W. F	Teenage	33	2	7.0	Del ex. 46-55 (out-of-frame)	14.2	12.3	1	Normal
20	50	DMD	258	W	48 yrs	43	3	2.6	Del ex. 14-21 (out-of-frame)	12.8	12.8	3	Normal
21	37	BMD	170	No	N/A	26	3	1.8	Del ex. 51 (out-of-frame)	15.7	13.3	2	Normal
22	45	BMD	274	P	37 yrs	24	3	2.3	c.5632C>T. p.(Gln1878^*^)	8.7	9.9	0	Decreased
23	56	BMD	470	W	47 yrs	47	1	2.9	Del ex. 45-48 (in-frame)	53.2	34.2	14	Decreased
24	41	DMD	146	P	39 yrs	34	3	2.8	Del ex. 46-50 (out-of-frame)	15.2	12.6	3	Normal
25	64	DMD	106	No	N/A	54	2	2.3	Del ex. 44 (out-of-frame)	16.9	13.9	7	Normal
26	76	DMD	112	W. P	N/A	25	1	3.9	Del ex. 48-52 (out-of-frame)	31.3	21.0	13	Decreased
27	45	DMD	77	P	37 yrs	-	3	3.7	Del ex. 45-48 (in-frame)	13.2	13.3	3	Decreased
28	41	BMD	99	No	N/A	20	3	5.1^*^	Del ex. 45-47 (in-frame)	11.6	9.4	1	Normal
29	52	DMD	1650	W	46 yrs	22	1	5.0^*^	Del ex. 47-51 (in-frame)	57.6	53.0	15	Decreased
30	33	BMD	165	No	N/A	12	3	3.2	Del ex. 45-48 (in-frame)	11.2	9.8	0	Normal
31	64	BMD	279	W. P	55 yrs	40	3	6.1^*^	Del ex. 45-49 (in-frame)	13.7	12.7	3	Decreased
32	55	DMD	739	W. P	Childhood	35	1	5.0^*^	Dup ex. 8-18 and 41-45 (out-of-frames)	70.8	63.7	15	Decreased
33	46	BMD	246	No	N/A	16	3	5.1^*^	Del ex. 13 (in-frame)	14.6	10.9	3	Decreased
34	29	DMD	255	W. P	N/A	27	3	4.3^*^	c.1114.delG	22.7	21.6	14	Decreased
35	44	BMD	114	No	N/A	28	3	1.8	Del ex. 45-47 (in-frame)	13.0	10.3	0	Normal
36	34	BMD	231	No	N/A	-	3	1.7	Del ex. 13 (in-frame)	16.4	12.9	4	Normal
37	69	BMD	241	No	N/A	47	3	1.0	Del ex. 45-47 (in-frame)	22.0	18.8	12	Normal
38	35	BMD	126	No	N/A	13	3	3.0	Del ex. 45-47 (in-frame)	10.4	9.8	0	Normal
39	38	BMD	277	No	N/A	25	3	4.6^*^	Del ex. 45-47 (in-frame)	12.7	10.6	0	Normal
40	33	DMD	1130	W. F	Childhood	27	2	5.3^*^	c.10108C>T. p.(Arg3370^*^)	23.2	19.8	9	Normal
41	52	DMD	66	No	N/A	32	3	5.3^*^	Del ex. 53-55 (out-of-frame)	14.4	11.1	2	Normal
42	57	DMD	747	W	N/A	43	3	2.6	Dup ex. 18-19 (out-of-frame)	19.5	19.5	11	Normal
43	40	DMD	188	W	Childhood	9	3	4.7^*^	c.3151C>T. p.(Arg1051^*^)	13.4	12.9	2	Normal
44	37	DMD	267	No	N/A	15	3	5.4^*^	Del ex. 45 (out-of-frame)	8.8	8.9	1	Normal
45	51	DMD	116	No	N/A	32	3	1.2	Dup ex. 44 (out-of-frame)	10.3	8.9	0	Normal
46	67	BMD	117	No	N/A	34	3	2.8	Del ex. 18-44 (out-of-frame)	20.1	13.7	6	Decreased
47	47	DMD	622	P	47 yrs	32	3	4.9^*^	c.6955C>T. p.(Gln2319^*^)	9.9	8.3	0	Normal
48	40	DMD	133	P	38 yrs	16	3	5.9^*^	c.8038C>T. p.(Arg2680^*^)	13.8	11.1	1	Normal
49	44	DMD	440	No	N/A	37	3	2.7	c.3295C>T. p.(Gln1099^*^)	10.9	10.8	0	Normal
50	33	DMD	877	P. F	33 yrs	33	3	2.6	Del ex. 51 (out-of-frame)	9.5	9.1	0	Decreased
51	57	BMD	79	No	N/A	26	3	1.9	Del ex. 44-48	12.7	8.8	0	Normal
52	81	DMD	541	No	N/A	30	2	3.4	Del ex. 53 (out-of-frame)	26.6	23.8	12	Decreased
53	42	DMD	126	W. F	Childhood	13	2	5.8^*^	Del ex. 44 (out-of-frame)	9.7	9.6	0	Decreased

† *Female carriers of pathogenic DMD variants predicted to cause Duchenne muscular dystrophy (DMD) or Becker muscular dystrophy (BMD)*.

†† *Blood creatine kinase normal reference range 35–210 U/l*.

††† *Self-reported, non-validated questionnaire, when asked: “Have you experienced any muscle symptoms i.e. muscle pain or weakness” W, weakness; P, pain; F, easily muscle fatigued*.

†††† *Physical function score, self-reported on non-validated questionnaire: 3) not affected (can run up a flight of stairs), 2) cannot run, but can walk up a flight of stairs without banisters, 1) can only walk upstairs by the help of banisters or a person, 0) cannot manage stairs*.

††††† *The mean fatigue severity score, FSS, in healthy subjects is 3.00 ± 1.08, and <4 is considered normal*.

* *marks ≥4 points. Min: 1, max: 7*.

♦ *List of the pathogenic genetic variants in the DMD gene (NM_004006.2) for each of the heterogenic carrier*.

♦♦ *Bilateral mean intramuscular fat fraction in thighs*.

♦♦♦ *Bilateral mean intramuscular fat fraction in calves*.

♦♦♦♦ *Number of abnormal muscles out of the total 16 muscle groups investigated in regard to fat fraction, compared to controls, that is, >2SD from control mean*.

♦♦♦♦♦ *Leg dynamometry performance compared to controls. “Decreased” refers to lower strength results than <2SD from control mean in one or more exercises (exercises being flexion and extension bilaterally of knees and ankles)*.

#### Physical Function

Eighteen women said they experienced weakness (Table 1). All participants were ambulatory. Seven women, all carriers of DMD-associated variants, reported difficulties running upstairs, but they could all climb the stairs without assistance (Table 1). Five women, of whom two carried BMD- and three DMD-associated variants in *DMD*, reported inability to climb the stairs without banisters or personal assistance. It should be noted that these five women all had BMI above 25 kg/m^2^, and one had a BMI of 42. Subjectively experiencing weakness tended to relate to higher fat fraction and weaker objective strength performance.

#### Pain

Thirteen of the 53 participants reported myalgia or cramps to some extent (Table 1). Pain complaints were not related to levels of muscle fat fraction or strength.

#### Fatigue

The mean fatigue score was 3.4 ± 1.55 (mean ± SD). Valko et al. measured the mean score among 454 healthy controls to be 3.0 ± 1.1 and defined a cutoff score for fatigue at ≥4 points (25). Nineteen women (36%) in our cohort scored ≥4 points with a dominance of carriers of DMD-associated variants (15 of 19) compared to 18% scoring ≥4 points in the 454 healthy controls (Table 1) (25). A higher FSS score did not correlate with higher fat fraction or weaker strength performance (Table 1).

## Discussion

In this observational, cross-sectional study of 53 women with pathogenic *DMD* variants, we used quantitative MRI and stationary dynamometry to describe skeletal muscle affection, which has not to our knowledge been studied before in this condition. Previous studies have, according to a review by Ishizaki et al. from 2018, investigated carriers' muscle strength by manual muscle testing, or handheld dynamometry only (13), and MRI studies of this group are rare and have assessed muscle involvement by using qualitative or semiquantitative methods (26–28). The findings of our study are that *DMD* variant carriers, who were selected based on their genetic defect and not symptoms, have higher fat fraction and lower muscle strength in the lower extremities compared to controls. Furthermore, specific force (muscle contractility) was also impaired in variant carriers vs. controls. There was no difference in fat fraction and muscle strength between women with pathogenic *DMD* variants predicted to produce DMD and BMD in men, but plasma CK and myoglobin levels were higher and fatigue and physical dysfunction were more pronounced in carriers of DMD-associated variants.

Asymmetry of replacement of muscle by fat was quite common. In 21% of the carriers, fat fractions differed by 10–38 percentage points, most commonly seen in the peroneal, lateral gastrocnemius, soleus, and hamstring muscles (Figure 6), without a convincing left vs. right dominance pattern, and the same was true for strength measures. We did not investigate handedness and do not know if side differences relate to handedness; however, no pattern indicating a more pronounced one-sided affection was evident. Previous reports on female *DMD* variant carriers have not reported on asymmetry vs. handedness either. Six women had prominent asymmetry in both fat fraction and strength assessments and were also the most functionally affected and struggled with stairs. Asymmetry in muscle affection has previously been shown by MRI and strength assessments in both mild and moderately to severely affected *DMD* variant carriers (8, 27, 29).

In boys with DMD, the general proximal pattern of leg involvement predominantly includes the medial and anterior compartments of the thighs, with a relative sparing of the gracilis, sartorius, and semimembranosus muscles (30), and in calves predominantly involving the peroneal, medial gastrocnemius, and soleus muscles (31). In the milder Becker muscular dystrophy, symmetric affection is common, and similar muscles as affected in DMD are observed (17). The pattern of muscle involvement in *DMD* variant carriers has previously been described in a retrospective study by Tasca et al. (27) in a cohort of 12 women. They investigated the involvement pattern using semiquantitative grading with CT and MRI and found a similar involvement pattern to those described in patients affected by DMD and BMD, including sparing of the sartorius and gracilis muscles, and a variable involvement of the calves. They found equally affected anterior, medial, and posterior compartments of the thigh. In our study, we used Dixon MRI to quantify muscle fat fractions. Dixon MRI is an objective measurement that quantifies the muscle content on a continuous scale, and because of its observer independency we believe it to be more precise than semiquantitative methods. In line with previous findings, we found side asymmetry and sparing of sartorius and gracilis. However, in our cohort, using the quantitative assessment of fat replacement of muscle, we found a more pronounced involvement of the posterior thigh muscles compared to anterior and medial muscles. Since mapping groups of muscles, instead of individual muscles, generates smaller error, we mapped muscles together in groups (32). Sartorius and gracilis were therefore included in the anterior and medial thigh group, respectively, and the abovementioned sparing of these muscles is based solely on visual interpretation.

We found that carriers of *DMD* variants were weaker than healthy age-matched women in both thigh and calf muscles. Notable weakness (<2SD from control mean) assessed with stationary dynamometry was seen in 21 (40%) women equally distributed between DMD- and BMD-associated variant carriers. When asked about experienced weakness, 18 (34%) of the 53 carriers had complaints of weakness to some extent. In 12 of these women, the subjective experience of muscle weakness agreed with the objective strength measures (<2SD from control mean). Nine women who did not report symptoms of weakness had notable muscle weakness on testing.

Manual muscle testing according to the MRC scale was not useful in this cohort. Although it is a simple and feasible method to evaluate muscle weakness, it is very investigator-dependent and it is not sensitive to detect weakness at the high end of the scale (33). Most women in our study had normal MRC testing, which is in line with neither our dynamometry and MRI findings nor the subjects' complaints. The MRC tests rarely detected an asymmetric weakness pattern, which was picked up by dynamometry assessments in some cases.

Asymmetry in muscle strength was not investigated in our control group but has previously been reported in healthy younger (34) and older (35) women to differ by 8.6–27% between sides in the lower extremities. In our study, asymmetry was not statistically significant for strength, but in some carriers, we did see side differences. Six carriers had strength asymmetry in the dynamometry of more than 50% in one or more assessments. Notable asymmetry in fat fraction and/or strength tended to relate to weakness complaints. Thus, quantitative MRI is more sensitive to disclose asymmetric affection and most likely detects such differences before they become clinically evident on strength testing.

All carriers and controls were examined in the same clinic using the same hardware. However, potential limitations to our study could have been introduced by using different software for MRI analysis and several evaluators for strength testing and MR scans. The MRI evaluations were not blinded; however, the two investigators analyzed images of controls and carriers separately, which would lessen a potential open-label observer bias.

Neither age nor BMI were perfectly matched between *DMD* variant carriers and controls. Controls were slightly younger and slimmer. However, removing the oldest and heavier carriers from the results gave the same results, which suggest that the subtle differences in age and BMI did not influence study results.

Our specific muscle force calculations showed reduced contractile properties of the lean muscle mass in *DMD* variant carriers in knee flexion and even more so in plantar flexion. Contractile properties of lean muscle mass has been shown to be disrupted in both men with Becker muscular dystrophy (36) and boys with Duchenne muscular dystrophy (37). In knee extension and ankle dorsal flexion, contractility was similar to that of controls. We found no difference between contractile properties in carriers of DMD- vs. BMD-associated variants. With increasing lean muscle mass, the maximum strength performed increased in our subjects. The correlation between CCSA and peak torque in the 18 healthy female controls was not convincing, which is likely due to small sample size and more importantly a narrow range of strength. However, a linear relationship between cross-sectional area and peak muscle force in healthy muscle is well-known (36, 38).

Myalgia and cramps are repeatedly reported as a symptom in *DMD* variant carriers (8, 11, 15, 39–41). In our study, reported leg pain was not related to levels of leg muscle fat fraction in the carriers. Supporting this finding, studies on paraspinal fat fraction in healthy men and women (20) and in facioscapulohumeral muscular dystrophy patients (42) found no correlation between lumbar fat fraction and lower back pain.

We found no difference between carriers of DMD- and BMD-associated variants in fat fraction of leg muscles or on strength testing. However, carriers of DMD-associated variants had more complaints of limb weakness, pain, and fatigue and CK and myoglobin levels were higher than in BMD-associated carriers. DMD-associated variant carriers have been reported to be slightly more affected by muscle weakness than BMD-associated variant carriers (8).

X-chromosome inactivation has frequently been hypothesized to explain disease variability in women carrying pathogenic *DMD* variants (8, 43, 44), but some reports on blood and muscle DNA have not confirmed this (40, 45). X-chromosome activation differs among tissues and therefore must be assessed in muscle. The muscle biopsy procedure was not part of the protocol and was historically only performed in very few of the women in this study. We did however collect a single muscle biopsy from one of the most affected participants (subject no. 32). This biopsy's X-chromosome inactivation pattern was skewed (90% mutant vs. 10% wild type). With only one biopsy, no firm conclusions can be reached but the result does point toward the X-chromosome inactivation theory as a plausible explanation for disease variability and calls for further studies.

Previous retrospective studies on the muscular status of female *DMD* variant carriers often included subjects based on hospital records, which would favor the bias of including symptomatic individuals. By including subjects prospectively and based on genetic information, we aimed to avoid such bias. Still, there is a possibility for selection bias, because about half of the invited participants did not take part in the study. The possibility of these women predominantly regarding themselves as either asymptomatic or too symptomatic to participate must be considered, which could skew the results in either direction. Non-participants were therefore contacted by phone for a brief questionnaire on muscle symptoms. Overall, non-participants did not differ in clinical symptoms from participants, and we therefore think our cohort is representative of women with pathogenic *DMD* variants in general.

Despite the prospective inclusion of participants in our study, we still found a high rate of manifestations in the carriers investigated. Symptomatic skeletal muscle manifestations have been reported to range between 2.5 and 22% (6, 8, 11, 13, 14). In our study, 43 of the 53 carriers (81%) were “manifesting” in terms of either abnormal strength or fat fraction, or both. This high rate relates to the sensitive measuring tools used in the study, that is, isokinetic dynamometry and quantitative MRI. Still, 30 of the 43 manifesting carriers were also symptomatic, which gives a more than 50% rate of symptomatic *DMD* variant carriers. The variety of published prevalence of muscular involvement in this cohort of women depends on several factors, including sensitivity and precision of assessments tools, as well as definitions used for manifesting vs. not manifesting carriers. In a prospective study, the subjective scoring of symptoms could be biased, perhaps resulting in augmented self-reported symptoms, merely due to the participant's knowledge of the study aim and their carriership. Nevertheless, our objective assessment tools disclose that carriership of pathogenic *DMD* variants results in a high prevalence of measurable manifestations, which for the most part also render the person symptomatic.

We chose the cutoff for abnormal values at two standard deviations from control mean, as this is an accepted and commonly used cutoff level in many scientific studies. A limitation when examining multiple parameters is however the inevitable risk of some values being abnormal by chance. Since we examined 16 muscle groups and 8 strength exercises, this should be considered a possible bias. The abnormal findings (72% had abnormal MRI results and 40% had abnormal strength results), however, were much larger than what can be explained merely by chance.

There are limitations to our study when it comes to number of muscles investigated. We did not investigate upper extremities, or pelvic muscles, mainly due to the need to limit the amount of data collection. An MRI study of lower and upper extremities as well as pelvic muscles in men with Becker muscular dystrophy, whom we phenotypically consider to resemble female *DMD* variant carriers more than men with DMD, showed a more severe involvement of lower extremities compared to upper extremities and showed an equal or slightly less involvement of pelvic muscles compared to thigh muscles (17). We therefore believe that any muscle involvement in carriers would be detected by the muscles we investigated. Another limitation of our study is that we did not measure strength across the hip, which could have been informative, given that hip strength is important in proximal myopathies. However, not having full MRI data (psoas major muscle was not scanned) to compare strength measurements with would also pose a limitation, and hip strength assessed by Biodex has a poorer reliability than strength measurements across knees and ankles.

In conclusion, most women with pathogenic *DMD* variants are symptomatic or show signs of degenerative changes in lower-extremity muscles, sometimes with an asymmetric pattern of fat replacement of muscle, but severe symptoms, as found in men affected by BMD, are rare. Longitudinal studies in female carriers of pathogenic *DMD* variants are needed to follow the evolution of these changes. A more affected phenotype for carriers of the DMD-associated mutations—compared to carriers of the BMD-associated mutations—was suggested by higher CK elevations and more complaints of weakness, pain, and fatigue, while muscle strength and fat fractions were similar between the two carrier groups. This is possibly explained by variable X-chromosome inactivation patterns. The findings suggest a need for an increased awareness of potential skeletal muscle involvement in women with pathogenic *DMD* variants.

## Data Availability Statement

The raw data supporting the conclusions of this article will be made available by the authors, without undue reservation.

## Ethics Statement

The studies involving human participants were reviewed and approved by Danish Data Protection Agency and the National Committee of Health Research. The patients/participants provided their written informed consent to participate in this study.

## Author Contributions

JV contributed to conception and design of the study. FF drafted the original manuscript and contributed to interpretation of data. FF, TS, A-SE, NP, AA, and MD contributed with data acquisition and subject recruitment. Statistical analysis and data interpretation were performed by JD. All authors contributed to revising the manuscript critically for intellectual content.

## Conflict of Interest

The authors declare that the research was conducted in the absence of any commercial or financial relationships that could be construed as a potential conflict of interest.

## Publisher's Note

All claims expressed in this article are solely those of the authors and do not necessarily represent those of their affiliated organizations, or those of the publisher, the editors and the reviewers. Any product that may be evaluated in this article, or claim that may be made by its manufacturer, is not guaranteed or endorsed by the publisher.

## Abbreviations

AC: anterior calf compartment (tibialis anterior muscle and m.extensor digitorum longus)
BMD: Becker muscular dystrophy
CCSA: contractile cross-sectional area
CK: creatine kinase
DP: deep posterior calf compartment (mm. tibialis posterior, flexor digitorum longus, flexor hallucis longus)
DMD: Duchenne muscular dystrophy
FF: fat fraction
F.Fo: Freja Fornander, MD;
GM: medial gastrocnemius muscle
J.R.D.: Julia Rebecka Dahlqvist, MD
MRC: medical research council scale
MRI: magnetic resonance imaging
PER: lateral calf compartment (mm. peroneus longus et brevis)
pT: peak torque
SOL + GL: soleus muscle and the lateral gastrocnemius muscle.
